# Immediate Loading Full-Arch 3D-Printed Implant-Supported Fixed Rehabilitation: A Case Report with 24-Month Follow-Up

**DOI:** 10.3390/medicina60101614

**Published:** 2024-10-02

**Authors:** Márcio de Carvalho Formiga, Renato Fuller, Lavinia Cosmina Ardelean, Jamil Awad Shibli

**Affiliations:** 1 Post-Graduation Program in Oral Implantology, University of the Itajaí Valley, Km 207 BR 101, São José 88103-800, SC, Brazil; marciocformiga@gmail.com (M.d.C.F.); fullerrenato@gmail.com (R.F.); 2 Academic Department of Technology of Materials and Devices in Dental Medicine, Multidisciplinary Center for Research, Evaluation, Diagnosis and Therapies in Oral Medicine, “Victor Babes” University of Medicine and Pharmacy Timisoara, 2 Eftimie Murgu Sq, 300041 Timisoara, Romania; 3 Department of Periodontology and Oral Implantology, Dental Research Division, Guarulhos University, 1 Tereza Cristina Sq., Guarulhos 07023-070, SP, Brazil; jshibli@ung.br

**Keywords:** 3D printing, dental implants, oral rehabilitation, osseointegration, immediate loading, DMLS

## Abstract

Implant-supported immediate loading full-arch rehabilitation has been documented in the literature. More recently, computed surgical guides have frequently been used since they facilitate planning and performing surgical treatment without the need to raise a flap, thus reducing trauma and morbidity. This case report describes an immediate full-arch, fixed rehabilitation with full loading placed on four commercially available 3D-printed implants, with a 24-month follow-up. The implants were placed with the help of a digitally planned 3D-printed surgical guide. The provisional fixed prosthesis installed immediately was replaced after 3 months. At the time, the soft and hard tissue around the implants appeared stable, without signs of inflammation. The same situation was observed at the 24-month follow-up. Three-dimensional-printed implants seem to be a promising choice in this case. However, further clinical studies with longer follow-up periods are necessary to confirm their efficacy.

## 1. Introduction

Most recently, implants have been submitted to functional loading immediately after being placed, a procedure that has shown high success rates in both arches, thus reducing the number of surgical interventions, and time elapsed between the surgical and prosthetic stages [1]. Immediate loading is defined as the placement of a temporary implant-supported prosthesis up to 48 h after implant insertion. It is often used in full-arch implant-supported rehabilitations, with good long-term results [2,3,4]. Previous studies on full-arch treatments with immediate loading have concluded that the placement of four implants, either two anterior parallel implants and two distally tilted implants, or four parallel implants, showed a similar stress distribution on the surrounding bone of the distal implants [5,6,7]. According to these studies, the manner of placement did not influence the rates of implant survival, success, and marginal bone loss after 2 years [5], 3 years [6], and 9 years of function [7]. These findings have allowed surgeons to choose their preferred manner of placing implants, after considering the particular anatomical situation. Computer-guided protocols that enable operators to study and plan the best possible position in which to place these implants are a feasible therapeutic option for implant-supported rehabilitation [8], in both the maxilla [9] and mandible [10].

The conventional implant placement protocol requires incisions, and complete detachment of the mucosa and periosteum, followed by exposure of bone tissue for placing the implant in a single or two-stage approach. This results in a prolonged surgical time and uncomfortable post-operative healing for the patient due to pain, edema, and hematoma. Whereas computer-guided, flapless implant surgery offers the advantages of high-precision implant placement, shorter surgical time, less trauma and bleeding, and less post-operative inflammation, edema, and hematoma [11,12]. It is, however, a highly technique-sensitive procedure at all stages that begins with the virtual planning and concludes with the surgical operation, all of which need to be accurately performed. Hence, only skilled and properly trained clinicians should undertake this type of advanced treatment [13].

Although computer-guided flapless surgery with virtual planning and a prototype guide may result in minimum angular and linear deviations, its levels of safety and accuracy are considered acceptable [14,15]. Greater deviations and other complications, such as nerve injury may, however, occur in the case of untrained or novice clinicians [13]. To perform immediate loading, some authors claim that it is mandatory to obtain installation torque values higher than 32 Ncm to provide sufficient primary stability [16,17].

Another important point to be considered when planning for guided flapless surgery is the fact that implant success is highly dependent on the preservation of the crestal bone, essential for implant stability and function. Soft tissue coverage and proper position of the implant–abutment junction have been stated as key factors in peri-implant bone remodeling [18].

Usually, in regions with thin mucosa, a more subcrestal implant placement should be planned, compared to areas with medium or thick mucosa, to benefit the supracrestal tissue height formation, and prevent unwanted exposure of the implant surface [19,20], and to maintain the peri-implant tissue stability [21,22]. In addition, the subcrestally placed morse-taper implants with platform switching show less bone resorption compared to crestal-positioned implants [23]. As a result of the implant–abutment connection position, micro gaps, micromovements, and prosthetic stress next to the crestal bone tissue are being avoided [24]. Another strategy to obtain diminished bone resorption in platform-switched implants is the installation and non-removal of the final abutment at the time of implant placement. This may also result in less bone remodeling around tapered implants placed subcrestally [25,26].

The enhanced stability between the abutments and the implants leads to lower micro-movements and better internal sealing, with no pumping of crevicular fluids or bacterial contamination close to the surrounding bone. Thus, the probability of bone remodeling that could lead to bone resorption is diminished [27].

Implant macro- and micro-geometries are directly related to primary stability, and after the healing period, it is related to the maintenance of osseointegration [28]. The transition from primary stability to secondary stability is characterized by bone remodeling around the implant during the healing process. Subsequently, the process of osseointegration is influenced by the implant surface characteristics [29,30]. The implant surface microgeometry directly influences the retention of blood clots and migration of bone cells to stimulate the osseointegration process, resulting in greater bone/implant contact, especially in regions with low-density bone [31]. To improve the microgeometry, implant surfaces treated with direct laser-sintered titanium powder have previously been studied, and have suggested that osteoblasts are activated by direct contact with the sintered implant surface, leading to contact osteogenesis [32,33].

More recently, advances in digital technology and 3D printing have significantly transformed the workflow and elevated the success rate of dental implants. Layer-by-layer material deposition allows precise control of the shape and texture of implants and the distribution of the material, resulting in improved biological properties, osseointegration, and protein adsorption, when compared with traditional machined implants [34,35]. This implant manufacturing technique has proven to be efficient and with better conditions to be adapted to the elastic properties of the bone [36].

Clinical case studies have shown that 3D-printed implants produced by laser sintering of titanium powder have attained a success rate comparable with that of conventional implant manufacturing technologies [37]. However, the manufacture of 3D-printed implants is still faced with certain challenges, such as obtaining the best quality surface, dimensional accuracy, and mechanical properties, not to mention the higher costs involved [38].

We aim to report a clinical case of a full-arch rehabilitation, screw retained on four commercially available 3D-printed implants, placed by computer-guided surgery, and submitted to immediate loading, with a 24-month follow-up.

## 2. Case Report

This case report was prepared according to the Clinical Case Reporting Guideline (CARE) [39], and the patient provided a written term of free and informed consent to publish this case. Moreover, this study was conducted in compliance with the Declaration of Helsinki, and approved by the Guarulhos University Ethics Committee on Clinical Research (CEP-UnG protocol code number 6.147.316).

**Figure 1:**
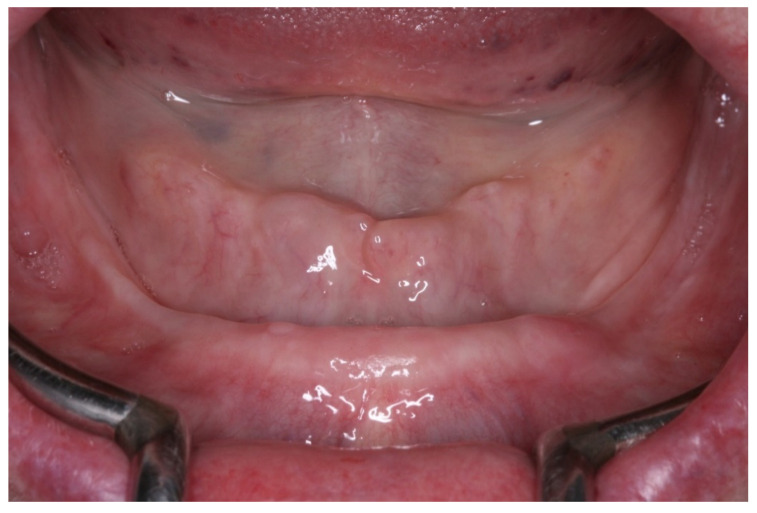
Initial view of the lower ridge mucosa (normal, healthy appearance).

The patient, a 72-year-old woman, with bi-maxillary complete dentures, came to our private practice complaining of discomfort with chewing, located in the left mental foraminal region. On inspection, the mucosa had a healthy appearance (Figure 1). The patient had recently replaced her old pair of dentures, and the new pair was aesthetically and functionally satisfactory, with correct vertical dimensions, occlusion, and good retention. Therefore, she was seeking an alternative solution to rehabilitation that would not compress the nerve emergence and provide her with comfort when chewing.

**Figure 2:**
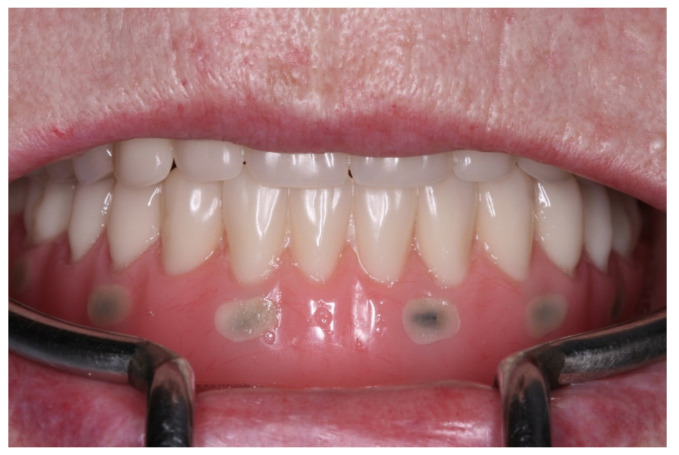
The lower denture with radiopaque marks to guide digital planning.

**Figure 3:**
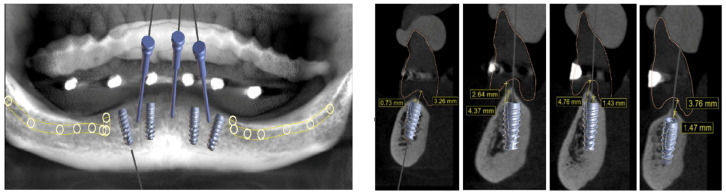
Establishing the position of the implant fixation pins; view of each implant position.

**Figure 4:**
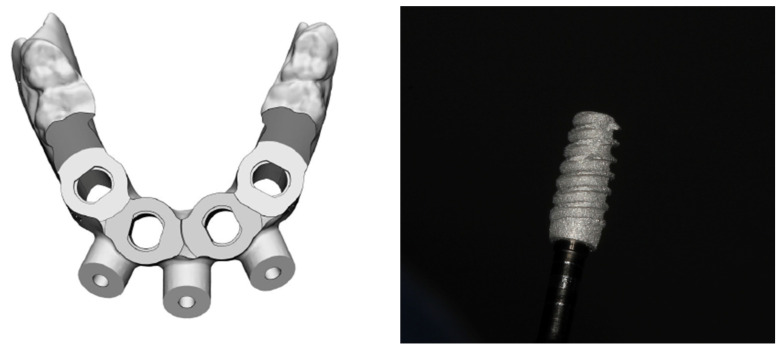
Virtual design of the computed surgical guide; RE 3D-printed 4.0 × 10 mm implant.

In a review of the medical history, the patient notified us that she had been using a prescribed anticoagulant due to a genetic heart disease. The clinical examination revealed the possible emergence of the alveolar nerve on the mandible crest, and the choice of full-arch rehabilitation with the flapless placement of four 3D-printed implants, with immediate loading was proposed and accepted by the patient. A computed tomography (CT) scan with the dentures in place was recommended and performed at the radiological examination center (Florianópolis, SC, Brazil). Six radiopaque marks were placed on the lower denture (Figure 2). Their role was to guide the digital planning of the best implant position. Furthermore, a computed surgical guide was planned, to avoid incisions and therefore reduce the risk of excessive bleeding during surgery, taking into consideration the patient’s systemic condition. The CT images were sent to the Plenum^®^ Bioengenharia planning center (Jundiaí, SP, Brazil), where the implant position was determined with the aid of Blue Sky Plan software (Blue Sky Bio, Libertyville, IL, USA) and the surgical guide was subsequently 3D printed. Four implants were planned, two anterior in an axial position and two slightly distal, at a safe distance from the mental foramen. In addition, three fixation pins were planned to eliminate the micro-movements of the computed surgical guide during use and thus minimize the possibility of errors (Figure 3 and Figure 4). The same company commercially produced the implants by additive manufacturing, namely direct metal laser sintering (DMLS) technology, using titanium powder grade 23. Although this type of implant has been recently launched to the market, a number of clinical studies have shown their efficacy, either in autologous [40] or synthetic biomaterial grafted sites [41].

**Figure 5:**
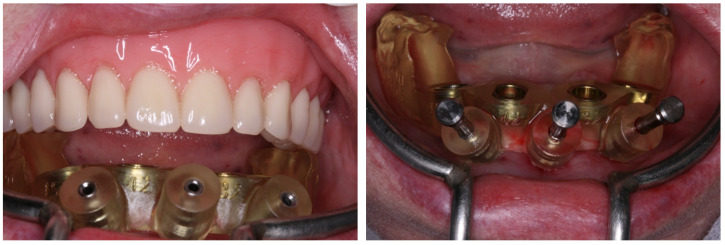
Testing the adaptation and stability of the computed surgical guide; the computed surgical guide is fixed to the mandible with fixation pins.

Three implant platforms, slim SL, regular RE, and short SH, with different dimensions are available. All implants have morse-taper internal connections, irrespective of the dimensions. The RE 4.0 × 10 mm type of implant was chosen for this case (Figure 4). Adaptation of the computed surgical guide was tested before the scheduled surgery appointment (Figure 5).

**Figure 6:**
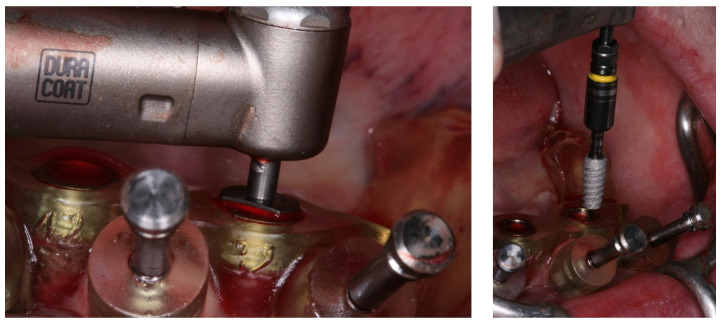
Bone instrumentation with the use of the computed surgical guide; implant placement with the use of the computed surgical guide.

**Figure 7:**
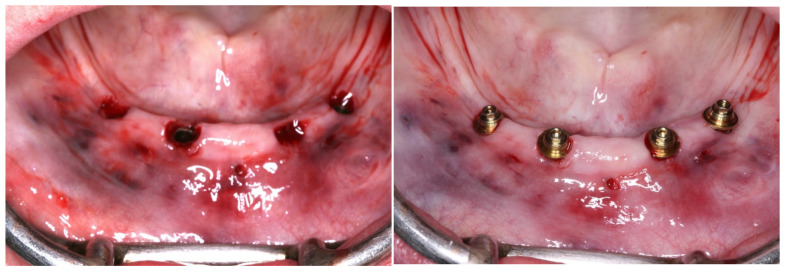
View of the mucosa immediately after implant placement and removal of the surgical guide; view of the mini-conical abutments installed.

**Figure 8:**
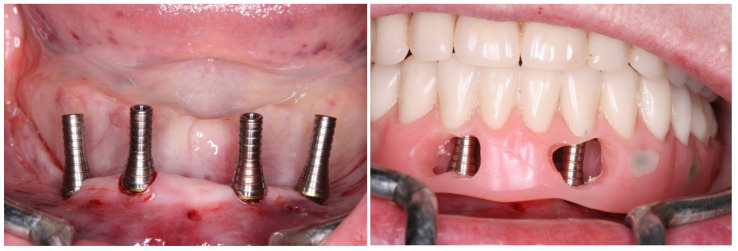
View of the titanium cylinders, screw retained to the abutments; denture preparation for capturing implant position.

**Figure 9:**
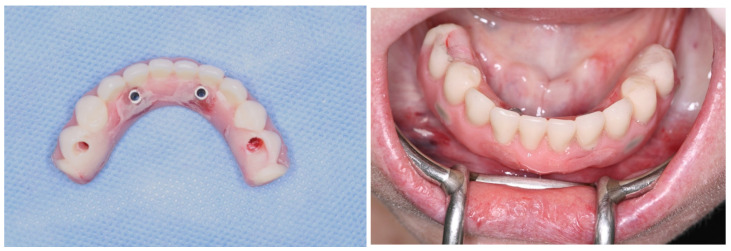
Lower denture transformed into a full-arch fixed screw-retained provisional prosthesis after modifications; view of the full-arch fixed screw-retained provisional prosthesis installed.

The preoperative medication consisted of 2 g of amoxicillin, one hour before the procedure, as suggested by the cardiologist. On the day of surgery, the patient received anesthesia with articaine 4% with epinephrine 1:100.000 (DFL, Rio de Janeiro, Brazil), the computed surgical guide was placed in position (assured by the occlusal position on both sides, and the stability over the patient’s mucosa), and immobilized with the fixation pins (Figure 5). The guided bone instrumentation protocol recommended by the implant manufacturer was followed, and four RE 4.0 × 10 mm implants (Figure 4) with a morse-taper connection were placed, without raising a flap (Figure 6). An insertion torque of 45 to 60 N/cm was used, ensuring the possibility of immediate load. Very little bleeding was observed during the surgery. The guide was removed and four mini-conical abutments, 2 mm high, produced by the same manufacturer, were installed on the implants (Figure 7). No sutures were necessary and minimal trauma in the soft tissue was noted (Figure 7). On these abutments, titanium cylinders were installed (Figure 8) and the lower denture was prepared to capture the implant position, transforming the removable prosthesis into a fixed type (Figure 8 and Figure 9). Pattern resin, characterized by high viscosity and low distortion, was used to join the denture to the cylinders, and pink acrylic resin was used to improve the esthetic appearance of the prosthesis. The denture was adjusted so that it caused no compression on the mucosa after it was screw retained to the implants. To diminish the cantilever, the molars on both sides of the denture were removed (Figure 9). The provisional full arch fixed screw-retained rehabilitation (Figure 9) was installed and enabled the patient to leave the dental surgery with an immediately functional loaded implant rehabilitation. The post-operative medication consisted of 600 mg of ibuprofen, twice a day, for 3 days if necessary. The patient was instructed to put ice bags on the surgical area for 48 h, for about 15 min every hour, not to go to sleep with the opposing complete denture in place for 7 days, and to eat only soft foods. On the day after surgery, the patient suspended the medication because she had no pain.

**Figure 10:**
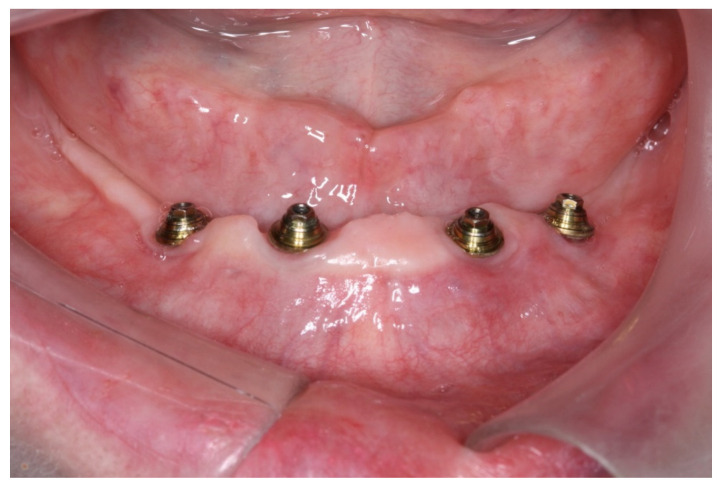
View of the peri-implant mucosa at 3 months after surgery.

**Figure 11:**
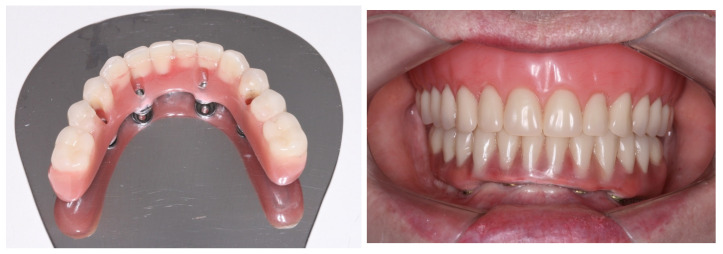
The new full-arch fixed screw-retained rehabilitation: extraoral lingual view; intraoral buccal view in occlusion.

**Figure 12:**
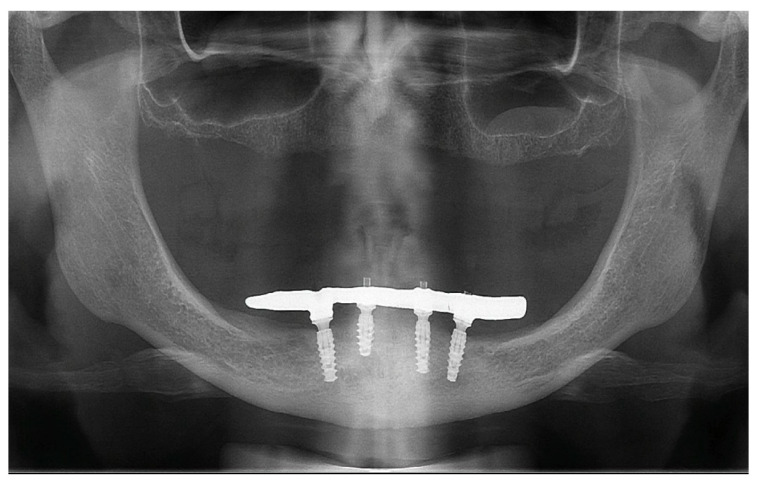
Panoramic radiograph at 24-month follow-up.

After a 3-month healing period, the patient returned to change the provisional fixed prosthesis. After removal of the provisional prosthesis, the peri-implant mucosa was noted to be healthy (Figure 10), without bleeding on probing. The peri-implant sulcus depth was lower than 3 mm, even in surrounding areas with no satisfactory keratinized tissue band, showing that the patient was able to maintain proper hygiene of the area underlying the prosthesis. A new, more delicate fixed rehabilitation was manufactured, with a reinforced metal bar for better ferulization of the implants and better dissipation of the stress during function (Figure 11). At the 24-month follow-up, the control panoramic radiograph showed no signs of bone remodeling around the implants (Figure 12), and the fixed prosthesis showed no sign of degradation.

## 3. Discussion

The “All-on-4” implant concept, which provides immediate, permanent, screw-retained, rehabilitation, is a well-established alternative treatment for edentulous patients [42,43]. Its excellent long-term results are backed up by numerous studies [3,4,44]. In this case, with the patient’s consent, this choice of treatment for the lower jaw rehabilitation was selected. The distal implants were not tilted as much as the original “All-on-4 concept” indicates. In line with previous studies that emphasized that parallel implants proved to be as effective as the tilted types [5,6,7], the 24-month follow-up of the present case indicated a successful outcome. The patient’s age did not seem to interfere with the result. Nevertheless, due to her systemic condition [45,46,47], a flapless computed-guided surgery was preferred, promoting greater comfort during and after the procedure [12,13,14,15]. The flapless computed-guided implant surgery has previously proven its accuracy and good clinical outcome, thus encouraging us to use it in this clinical case [12,15,48].

Advances in digital implantology have not been limited to planning implant positions, producing computed surgical guides, or preparing prosthetic rehabilitation even before surgery, with reliable accuracy [49,50,51]. More recently, 3D-printed custom-made dental implants, made of titanium or other bio-compatible materials, such as zirconium and polyether-ether-ketone (PEEK), gained widespread attention [52,53]. Titanium is still considered the best material for dental implantology, because of its mechanical resistance, biocompatibility, osseointegration, and anti-bacterial properties [54].

This case report described the placement of commercially available titanium dental implants produced by additive manufacturing. To the best of our knowledge, this is the only 3D-printed commercially available implant system, providing the choice of three morse-taper implant platforms (slim SL, regular RE, short SH), with an 11° angulation for the RE and SL types and 15° for the SH types. The most appropriate implant dimension may be chosen from a large range of lengths (from 5.0 to 15.0 mm) and diameters (from 3.0 to 6.0 mm), depending on the platform. The versatility of the system is improved by color coding. Their progressive thread geometry, with active spirals, causes less surgical trauma and provides better primary stability [55].

The layer-by-layer build-up of 3D-printed implants offers versatility, accuracy, and improved mechanical properties [36,56] and it enables better control of the implant micro and nano-geometry. The implant can be constructed with a functional porosity gradient, which makes it possible to balance the difference between the elastic moduli of bone tissue and the titanium implant. This results in reduced stress on functional load and provides long-term tissue stability [34]. Three-dimensional-printed implants exhibit rough surfaces, which promote osteogenic differentiation and osseointegration [57].

According to a recent systematic review of clinical trials, rough surfaces and loaded implants display better histological healing outcomes [58]. Another recent review to analyze different implant surfaces and identify the ideal structure from a clinical and durability point of view concluded that 3D-printed implants deserved further investigation [52].

A recent animal study compared the sequential osseointegration of titanium 3D-printed implants with conventional types. Due to the 3D network of the trabecular structure of the 3D-printed implants, the total surface available for the osseointegration process was higher than that of the conventional implants, shown by higher bone-to-implant contact. The authors concluded that the 3D-printed implants were successfully osseointegrated, with adequate fractions of mineralized bone formation after 2 and 6 weeks [59].

The titanium implants used in the present case report are produced by DMLS, which represents a more recent advancement in 3D-printing powder bed fusion technology and has proven to significantly improve the implant reliability, accuracy, success rates, osseointegration levels, and long-term efficacy [32,60]. DMLS is considered to have significantly simplified the process when compared with traditional methods process and has emerged as a potential manufacturing technique for the production of different types of implants [61,62,63,64].

An in vitro study [65], evaluating the influence of conventional and DMLS implant production on the microbial adhesion of periodontal pathogens, showed that DMLS could modify the biofilm profile by reducing the proportion of red complex species and decreasing total counts of *Porphyromonas gingivalis*, known to be related to peri-implant disease.

According to the manufacturer (Plenum^®^ Bioengenharia, Jundiaí, SP, Brazil), the 3D-printed implants are characterized by a surface that mimics the trabeculated bone structure, with controlled wettability, which permits the concentration of growth factors and facilitates cell adhesion [55]. This statement was confirmed by a recent in vitro study that evaluated the interaction between five different implant surfaces and fibrinogen, as the first step for the formation of the fibrin network. Among the five commercially available implants assessed, the type of implant used in this clinical case report was included. The study concluded that its surface had the best performance on coverage by liquid fibrinogen, with a thick and dense fibrin layer with more cells trapped inside. According to the authors, the topographic characteristics of this implant, such as mimicking the microstructure of a trabecular bone, may stimulate cell adhesion and new bone formation due to the thicker fibrin fibers inserted and interconnected between its microroughness [66].

Because of their novelty, information on the clinical outcomes of this type of commercially available 3D-printed implants is scarce. The only reference we could find belongs to da Rocha Scalzer Lopes et al., who successfully treated a case of upper left premolars agenesis by installing two commercially available 3D-printed implants (Plenum^®^ Bioengenharia, Jundiaí, SP, Brazil), in association with maxillary sinus floor lift with synthetic regenerative materials. They described it as a promising option in areas of deficient bone. They also stated that the association of the two techniques significantly reduced the treatment time [67].

Despite all the advantages of flapless surgery, it does not consider the soft tissue quality, and in certain situations, as in our case, some of the implants may be placed in areas with little or no keratinized tissue. It is well documented that the presence of keratinized tissue around implants has a positive impact on the health and stability of peri-implant tissues [68], while its absence is associated with an increased risk of mucosal inflammation [69].

Due to the patient’s systemic conditions, performing another surgery to increase soft tissue width or thickness was not supported by the patient’s attending physician, thus requiring closer at-home and professional biofilm control.

## 4. Conclusions

This case report describes a successful immediately loaded restoration protocol, supported by four surgically guided 3D-printed implants. This new commercially available type of implant is produced by additive manufacturing and showed no biological or mechanical complications at a 24-month follow-up.

Compared to custom-made implants, commercially available 3D-printed implants bring the advantage of reproducibility and controlled manufacturing, which may reduce stress on functional load. The fact that they are ready to use may result in a shortened workflow and reduced treatment time [67]. Compared to other commercially available dental implants, with surfaces obtained by specific manufacturing techniques, the 3D-printed commercially available implants used in this case are characterized by an organic, homogeneous surface design, which mimics the microstructure of the bone trabeculae [55,66,67]. To date, as stated by the limited publications available, on a micro/nanotopographic scale, their surface displays a high surface energy and an improved wettability, thus favoring the diffusion and adhesion of fibrin and protein matrix, modulating cell behavior, and stimulating cell proliferation and differentiation [66,67]. Compared to other implants with nanotextured but heterogeneous surfaces, the homogeneous surface of the implants used in this case plays a role in accelerated osseointegration, especially in areas where bone neoformation is more sensitive [67].

Despite the promising results available so far [66,67], further controlled clinical trials must be conducted to assess their medium- and long-term results, focusing on peri-implant mucosa health and bone resorption.

## Data Availability

The original contributions presented in this study are included in the article, further inquiries can be directed to the corresponding author.

## References

[1] Del Fabbro M., Testori T., Kekovic V., Goker F., Tumedei M., Wang H.L. (2019). A systematic review of survival rates of osseointegrated implants in fully and partially edentulous patients following immediate loading. J. Clin. Med..

[2] Gonçalves G.S.Y., de Magalhães K.M.F., Rocha E.P., Dos Santos P.H., Assunção W.G. (2022). Oral health-related quality of life and satisfaction in edentulous patients rehabilitated with implant-supported full dentures all-on-four concept: A systematic review. Clin. Oral Investig..

[3] Agliardi E.L., Pozzi A., Romeo D., Del Fabbro M. (2023). Clinical outcomes of full-arch immediate fixed prostheses supported by two axial and two tilted implants: A retrospective cohort study with 12-15 years of follow-up. Clin. Oral Implants Res..

[4] Malò P., De Araujo Norbre M., Lopes A., Ferro A., Botto J. (2019). The All-on-4 treatment concept for the rehabilitation of the completely edentulous mandible: A longitudinal study with 10 to 18 years of follow-up. Clin. Implant. Dent. Relat. Res..

[5] Badr S., Elawady D., Ibrahim W., Eldin A. (2023). Axial versus tilted distal implants in All-on-4 mandibular screw-retained prosthesis. A randomized controlled trial. MSA Dent. J..

[6] Mehta S.P., Sutariya P.V., Pathan M.R., Upadhyay H.H., Patel S.R., Kantharia N.D.G. (2021). Clinical success between tilted and axial implants in edentulous maxilla: A systematic review and meta-analysis. J. Indian. Prosthodont. Soc..

[7] Tironi F., Orlando F., Azzola F., Corbella S., Francetti L.A. (2022). A retrospective analysis on marginal bone loss around tilted and axial implants in immediate-loaded All-On-4 with a long-term follow-up evaluation. Prosthesis.

[8] Cattoni F., Chirico L., Merlone A., Manacorda M., Vinci R., Gherlone E.F. (2021). Digital smile designed computer-aided surgery versus traditional workflow in “All on Four” rehabilitations: A randomized clinical trial with 4-years follow up. Int. J. Environ. Res. Public Health.

[9] Manacorda M., Poletti de Chaurand B., Merlone A., Tetè G., Mottola F., Vinci R. (2020). Virtual implant rehabilitation of the severely atrophic maxilla: A radiographic study. Dent. J..

[10] Miljanovic D., Seyedmahmoudian M., Horan B., Stojcevski A. (2022). Novel and accurate 3D-printed surgical guide for mandibular reconstruction with integrated dental implants. Comput. Biol. Med..

[11] D’Haese J., Ackhurst J., Wismeijer D., De Bruyn H., Tahmaseb A. (2017). Current state of the art of computer-guided implant surgery. Periodontol. 2000.

[12] Naeini E.N., Atashkadeh M., De Bruyn H., D’Haese J. (2020). Narrative review regarding the applicability, accuracy, and clinical outcome of flapless implant surgery with or without computer guidance. Clin. Implant. Dent. Relat. Res..

[13] Unsal G.S., Turkyilmaz I., Lakhia S. (2020). Advantages and limitations of implant surgery with CAD/CAM surgical guides: A literature review. J. Clin. Exp. Dent..

[14] Cunha R.M., Souza F.A., Hadad H., Poli P.P., Maiorana C., Carvalho P.S.P. (2021). Accuracy evaluation of computer-guided implant surgery associated with prototyped surgical guides. J. Prosthet. Dent..

[15] La Monaca G., Pranno N., Annibali S., Di Carlo S., Pompa G., Cristalli M.P. (2022). Immediate flapless full-arch rehabilitation of edentulous jaws on 4 or 6 implants according to the prosthetic-driven planning and guided implant surgery: A retrospective study on clinical and radiographic outcomes up to 10 years of follow-up. Clin. Implant. Dent. Relat. Res..

[16] Atieh M.A., Baqain Z.H., Tawse-Smith A., Ma S., Almoselli M., Lin L., Alsabeeha N.H.M. (2021). The influence of insertion torque values on the failure and complication rates of dental implants: A systematic review and meta-analysis. Clin. Implant. Dent. Relat. Res..

[17] Darriba I., Seidel A., Moreno F., Botelho J., Machado V., Mendes J.J., Leira Y., Blanco J. (2023). Influence of low insertion torque values on survival rate of immediately loaded dental implants: A systematic review and meta-analysis. J. Clin. Periodontol..

[18] Rossi R., Capri D., Risciotti E., Zeman P. (2015). Randomized Clinical Investigation of Titanium Implants with and without Platform Switching: Six Months’ Radiographic and Clinical Outcome. Dent. J..

[19] Spinato S., Bernardello F., Lombardi T., Soardi C.M., Messina M., Zaffe D., Stacchi C. (2022). Influence of apico-coronal positioning of tissue-level implants on marginal bone stability during supracrestal tissue height establishment: A multi-center prospective study. Clin. Implant. Dent. Relat. Res..

[20] Bambini F., Orilisi G., Quaranta A., Memè L. (2021). Biological oriented immediate loading: A new mathematical implant vertical insertion protocol, Five-year follow-up study. Materials.

[21] Degidi M., Perrotti V., Shibli J.A., Novaes A.B., Piattelli A., Iezzi G. (2011). Equicrestal and subcrestal dental implants: A histologic and histomorphometric evaluation of nine retrieved human implants. J. Periodontol..

[22] Jensen S.S., Aghaloo T., Jung R.E., Bertl K., Buser D., Chappuis V., de Stavola L., Monje A., Pispero A., Roccuzzo A. (2023). Group 1 ITI Consensus Report: The role of bone dimensions and soft tissue augmentation procedures on the stability of clinical, radiographic, and patient-reported outcomes of implant treatment. Clin. Oral Implant. Res..

[23] Veis A., Parissis N., Tsirlis A., Papadeli C., Marinis G., Zogakis A. (2010). Evaluation of peri-implant marginal bone loss using modified abutment connections at various crestal level placements. Int. J. Periodont. Rest. Dent..

[24] Alonso-González R., Aloy-Prósper A., Peñarrocha-Oltra D., Peñarrocha-Diago M.A., Peñarrocha-Diago M. (2012). Marginal bone loss in relation to platform switching implant insertion depth: An update. J. Clin. Exp. Dent..

[25] Degidi M., Nardi D., Piattelli A. (2011). One abutment at one time: Non-removal of an immediate abutment and its effect on bone healing around subcrestal tapered implants. Clin. Oral. Implant. Res..

[26] Wang Q.Q., Dai R., Cao C.Y., Fang H., Han M., Li Q.L. (2017). One-time versus repeated abutment connection for platform-switched implant: A systematic review and meta-analysis. PLoS ONE.

[27] Tomar S., Saxena D., Kaur N. (2023). Marginal bone loss around implants with platform switching and platform matched connection: A systematic review. J. Prosthet. Dent..

[28] Tumedei M., Piattelli A., Degidi M., Mangano C., Iezzi G. (2020). A narrative review of the histological and histomorphometrical evaluation of the peri-implant bone in loaded and unloaded dental implants. A 30-year experience (1988–2018). Int. J. Environ. Res. Public Health.

[29] Bergamo E.T.P., Zahoui A., Barrera R.B., Huwais S., Coelho P.G., Karateew E.D., Bonfante E.A. (2021). Osseodensification effect on implants primary and secondary stability: Multicenter controlled clinical trial. Clin. Implant. Dent. Relat. Res..

[30] Huang Y.C., Huang Y.C., Ding S.J. (2023). Primary stability of implant placement and loading related to dental implant materials and designs: A literature review. J. Dent. Sci..

[31] Coelho P.G., Granjeiro J.M., Romanos G.E., Suzuki M., Silva N.R., Cardaropoli G., Thompson V.P., Lemons J.E. (2009). Basic research methods and current trends of dental implant surfaces. J. Biomed. Mater. Res. B Appl. Biomater..

[32] Mangano C., Piattelli A., Raspanti M., Mangano F., Cassoni A., Iezzi G., Shibli J.A. (2011). Scanning electron microscopy (SEM) and X-ray dispersive spectrometry evaluation of direct laser metal sintering surface and human bone interface: A case series. Lasers Med. Sci..

[33] Mangano C., Raspanti M., Traini T., Piattelli A., Sammons R. (2009). Stereo imaging and cytocompatibility of a model dental implant surface formed by direct laser fabrication. J. Biomed. Mater. Res. A.

[34] Suh H., Lee D., Lee J., Seol Y.J., Lee Y.M., Koo K.T. (2023). Comparative evaluation of 3D-printed and conventional implants in vivo: A quantitative microcomputed tomographic and histomorphometric analysis. Sci. Rep..

[35] Iezzi G., Zavan B., Petrini M., Ferroni L., Pierfelice T.V., D’Amora U., Ronca A., D’Amico E., Mangano C. (2024). 3D printed dental implants with a porous structure: The in vitro response of osteoblasts, fibroblasts, mesenchymal stem cells, and monocytes. J. Dent..

[36] Traini T., Mangano C., Sammons R.L., Mangano F., Macchi A., Piattelli A. (2008). Direct laser metal sintering as a new approach to fabrication of an isoelastic functionally graded material for manufacture of porous titanium dental implants. Dent. Mater..

[37] Huang S., Wei H., Li D. (2023). Additive manufacturing technologies in the oral implant clinic: A review of current applications and progress. Front. Bioeng. Biotechnol..

[38] Oliveira T.T., Reis A.C. (2019). Fabrication of dental implants by the additive manufacturing method: A systematic review. J. Prosthet. Dent..

[39] Gagnier J.J., Kienle G., Altman D.G., Moher D., Sox H., Riley D., CARE Group (2013). The CARE guidelines: Consensus-based clinical case reporting guideline development. Glob. Adv. Health Med..

[40] Louro R.S., Moraschini V., Melhem-Elias F., Sturzinger G.P.S., Amad R.A., Shibli J.A. (2024). Digital implant-supported restoration planning placed in autologous graft using titanium implants produced by additive manufacturing. Dent. J..

[41] Mafra I.J., Bordin D., Siroma R.S., Moraschini V., Faverani L.P., Souza J.G., Mourão C.F., Shibli J.A. (2024). Additive manufacturing titanium dental implants placed in sinuses grafted with 70HA:30-TCP: A one-year retrospective study for evaluation of survival rate. Dent. J..

[42] Soyfer V. (2023). Review article on the All-On-Four treatment concept in dental implants. Arch. Surg. Clin. Res..

[43] Chan M.H., Nudell Y.A. (2021). All-on-4 concept update. Dent. Clin. N. Am..

[44] Maló P., de Araujo Nobre M., Lopes A., Ferro A., Nunes M. (2019). The All-on-4 concept for full-arch rehabilitation of the edentulous maxillae: A longitudinal study with 5-13 years of follow-up. Clin. Implant. Dent. Relat. Res..

[45] Corbella S., Agliardi E., Basso M., Romeo D., Testori T., Francetti L. (2008). Management of dental patients taking anticoagulant drugs. Dent. Cadmos.

[46] Kelly N., Beaton L., Knights J., Stirling D., West M., Young L. (2023). The practices and beliefs of dental professionals regarding the management of patients taking anticoagulant and antiplatelet drugs. BDJ Open.

[47] Tetè G., Polizzi E., D’orto B., Carinci G., Capparè P. (2021). How to consider implant-prosthetic rehabilitation in elderly patients: A narrative review. J. Biol. Regul. Homeost. Agents.

[48] Subramani K. (2022). Is computer-guided implant placement with a flapless approach more accurate than with a flapped surgical approach?. Evid. Based Dent..

[49] Tallarico M. (2020). Computerization and digital workflow in medicine: Focus on digital dentistry. Materials.

[50] Todaro C., Cerri M., Isola G., Manazza A., Storelli S., Rodriguez y Baena R., Lupi S.M. (2023). Computer-guided osteotomy with simultaneous implant placement and immediately loaded full-arch fixed restoration: A case report. Prosthesis.

[51] Todaro C., Cerri M., Rodriguez Y., Baena R., Lupi S.M. (2023). Full-arch guided restoration and bone regeneration: A complete digital workflow case report. Healthcare.

[52] Inchingolo A.M., Malcangi G., Ferrante L., Del Vecchio G., Viapiano F., Inchingolo A.D., Mancini A., Annicchiarico C., Inchingolo F., Dipalma G. (2023). Surface Coatings of Dental Implants: A Review. J. Funct. Biomater..

[53] Sonaye S.Y., Bokam V.K., Saini A., Nayak V.V., Witek L., Coelho P.G., Bhaduri S.B., Bottino M.C., Sikder P. (2022). Patient-specific 3D printed Poly-ether-ether-ketone (PEEK) dental implant system. J. Mech. Behav. Biomed. Mater..

[54] Safi I.N., Hussein B.M.A., Aljudy H.J., Tukmachi M.S. (2021). Effects of long durations of RF–magnetron sputtering deposition of hydroxyapatite on titanium dental implants. Eur. J. Dent..

[55] Plenum Bioengeharia. https://plenum.bio/en/products/implants.

[56] Goguta L., Lungeanu D., Negru R., Birdeanu M., Jivanescu A., Sinescu C. (2021). Selective laser sintering versus Selective laser melting and Computer aided design—Computer aided manufacturing in double crowns retention. J. Prosthodont. Res..

[57] Lee J., Lee J.B., Yun J., Rhyu Y.C., Lee Y.M., Lee S.M., Lee M.K., Kim B., Kim P., Koo K.T. (2021). The impact of surface treatment in 3-dimensional printed implants for early osseointegration: A comparison study of three different surfaces. Sci. Rep..

[58] Chambrone L., Rincón-Castro M.V., Poveda-Marín A.E., Diazgranados-Lozano M.P., Fajardo-Escolar C.E., Bocanegra-Puerta M.C., Palma L.F. (2020). Histological healing outcomes at the bone-titanium interface of loaded and unloaded dental implants placed in humans: A systematic review of controlled clinical trials. Int. J. Oral Implantol..

[59] Lang N.P., Imber J.C., Lang K.N., Schmid B., Muñoz F., Bosshardt D.D., Saulacic N. (2023). Sequential osseointegration of a novel implant system based on 3D printing in comparison with conventional titanium implants. Clin. Oral Implant. Res..

[60] Mangano F., Mangano C., Piattelli A., Iezzi G. (2017). Histological evidence of the osseointegration of fractured direct metal laser sintering implants retrieved after 5 years of function. Biomed Res. Int..

[61] Łoginoff J., Majos A., Elgalal M. (2024). The evolution of custom subperiosteal implants for treatment of partial or complete edentulism in patients with severe alveolar ridge atrophy. J. Clin. Med..

[62] Dantas T., Vaz P., Samuel F. (2023). Subperiosteal dental implants: Past or future? A critical review on clinical trials/case reports and future directions. J. Dent. Implant..

[63] Mangano C., Bianchi A., Mangano F.G., Dana J., Colombo M., Solop I., Admakin O. (2020). Custom-made 3D printed subperiosteal titanium implants for the prosthetic restoration of the atrophic posterior mandible of elderly patients: A case series. 3D Print. Med..

[64] Onică N., Budală D.G., Baciu E.-R., Onică C.A., Gelețu G.L., Murariu A., Balan M., Pertea M., Stelea C. (2024). Long-term clinical outcomes of 3D-printed subperiosteal titanium implants: A 6-year follow-up. J. Pers. Med..

[65] Pingueiro J., Piattelli A., Paiva J., Figueiredo L.C., Feres M., Shibli J., Bueno-Silva B. (2019). Additive manufacturing of titanium alloy could modify the pathogenic microbial profile: An in vitro study. Braz. Oral Res..

[66] Andrade C.X., Quirynen M., Rosenberg D.R., Pinto N.R. (2021). Interaction between different implant surfaces and liquid fibrinogen: A pilot in vitro experiment. Biomed Res. Int..

[67] da Rocha Scalzer Lopes G., de Matos J.D.M., Oliveira D., Condé Oliveira Prado P.H., Garcia Rocha M., Coelho Sinhoreti M.A., Bottino M.A., de Carvalho Ramos N. (2022). Posterior rehabilitation using 3D-printed dental implants and synthetic regenerative biomaterials. Braz. Dent. Sci..

[68] Heydari M., Ataei A., Riahi S.M. (2021). Long-term effect of keratinized tissue width on peri-implant health status indices: An updated systematic review and meta-analysis. Int. J. Oral Maxillofac. Implant..

[69] Oh S.L., Shahami S., Bernal-Cepeda L.J., Fu Y., Chung M.K. (2024). Therapeutic effectiveness of keratinized mucosa augmentation for functioning dental implants: A systematic review and meta-analysis. J. Prosthodont. Res..

